# Solitary Testicular Metastasis From Ductal Prostate Cancer 5 Years After Radical Prostatectomy: A Case Report

**DOI:** 10.1155/criu/2532587

**Published:** 2026-05-25

**Authors:** Martina Romani, Tonino Morena, Denise Bianchi, Chiara Ogliosi, Alberto Pettenò, Alberto Zaniboni, Angelo Peroni

**Affiliations:** ^1^ Urology Unit Fondazione Poliambulanza Hospital, Brescia, Italy; ^2^ Urology Unit ASST Spedali Civili Hospital, University of Brescia, Brescia, Italy, brescia.edu; ^3^ Pathology Unit Fondazione Poliambulanza Hospital, Brescia, Italy; ^4^ Oncology Unit Fondazione Poliambulanza Hospital, Brescia, Italy

**Keywords:** case report, ductal prostate cancer, orchiectomy, radical prostatectomy, testicular metastasis, urologic oncology

## Abstract

Testicular metastases from prostate carcinoma are exceedingly uncommon, particularly from the ductal histological subtype, which is associated with aggressive clinical behavior and a propensity for atypical metastatic dissemination. We report the case of a 73‐year‐old male with a history of prostate ductal adenocarcinoma (PDA) treated in 2018 with robot‐assisted radical prostatectomy (RARP) and extended pelvic lymphadenectomy. Pathological staging was pT2c R0 N0 (0/53 nodes), and postoperative PSA was undetectable. The patient remained free of recurrence for 5 years. In March 2023, a biochemical recurrence prompted repeated thoracoabdominal computed tomography (CT) and pelvic magnetic resonance imaging (MRI) scans; neither study included dedicated scrotal imaging, and both were negative for recurrence. By March 2024, serum PSA had increased to 5.6 ng/mL, coinciding with the onset of progressive, painless enlargement of the left testicle. Scrotal ultrasonography revealed a solid mass replacing the left testicular parenchyma. 18F‐Fluorodeoxyglucose positron emission tomography/computed tomography (FDG PET/CT) and choline PET/CT showed a hypermetabolic lesion confined to the left testis, with no evidence of extratesticular disease. The patient underwent left inguinal orchiectomy. Histopathological examination revealed metastatic adenocarcinoma with papillary architecture, morphologically consistent with ductal prostate origin. PSA levels declined rapidly postoperatively, reaching 0.07 ng/mL within 40 days. At 13‐month follow‐up, the patient remained clinically and biochemically free of disease without further treatment. This case represents one of the few documented instances of solitary testicular metastasis from PDA. It underscores the importance of continued PSA monitoring and highlights that isolated testicular involvement, whereas rare, may confer a more favorable prognosis than typically expected in metastatic PDA. In selected cases, orchiectomy may serve both diagnostic and therapeutic roles, supporting a conservative postoperative approach.

## 1. Introduction

Prostatic ductal adenocarcinoma (PDA) represents the most prevalent histological variant of prostate cancer (PCa), with an estimated incidence ranging from 0.15% to 7% across reported series [1, 2]. Compared with conventional acinar adenocarcinoma (PAA), PDA exhibits more aggressive behavior, including a higher likelihood of advanced local disease at diagnosis, atypical sites of metastasis, and poorer prognosis [3–5]. Among these atypical sites, testicular metastases are exceedingly uncommon, with only a handful of cases described in the literature [6–9].

The testis is considered a sanctuary site due to physiological and anatomical factors such as the blood–testis barrier and lower scrotal temperature, which limit tumor implantation and proliferation [10–12]. When testicular metastases do occur, they are most frequently found incidentally during orchiectomy for androgen deprivation therapy or autopsy [13–15].

Here, we present a unique case of solitary testicular metastasis from PDA in a patient with no evidence of systemic disease 5 years after radical prostatectomy. The case calls attention to the diagnostic value of prostate‐specific antigen (PSA) kinetics, the importance of multimodal imaging, and the potential therapeutic role of orchiectomy in select patients with isolated recurrence.

## 2. Case Presentation

A 73‐year‐old male with a medical history of hypertension, obesity, and Gilbert′s syndrome was diagnosed with PCa in 2018 following an elevated PSA level of 6 ng/mL and an abnormal digital rectal examination. Multiparametric magnetic resonance imaging (mpMRI) revealed a PI‐RADS 5 lesion in the left basal peripheral zone. Targeted prostate biopsy confirmed ductal adenocarcinoma.

A staging choline positron emission tomography/computed tomography (PET/CT) scan demonstrated no evidence of metastatic disease. The patient underwent robot‐assisted laparoscopic radical prostatectomy (RARP) with extended pelvic lymph node dissection. Final histopathology revealed a ductal carcinoma, HGPIN‐like variant, Gleason score 3 + 3, staged as pT2c R0 N0 (0/53 nodes), cM0. This variant is characterized by architectural features resembling high‐grade prostatic intraepithelial neoplasia and is typically associated with lower Gleason grading compared with conventional ductal adenocarcinoma.

The postoperative PSA was undetectable, and no adjuvant therapy was administered.

In March 2023, progressive PSA elevation prompted two thoracoabdominal computed tomography (CT) scans and two pelvic MRIs, all of which were negative for recurrence or metastasis. The case was reviewed in a multidisciplinary meeting, where it was decided to defer radiotherapy.

By March 2024, PSA levels had reached 5.6 ng/mL. At this time, the patient reported a two‐month history of progressive, painless enlargement of the left testicle. Serum tumor markers (*α*‐fetoprotein, *β*‐human chorionic gonadotropin, and lactate dehydrogenase) were within normal limits. Physical examination revealed a firm, irregular enlargement of the left testis. Scrotal ultrasound demonstrated a solid mass replacing the testicular parenchyma (Figure 1). 18F‐Fluorodeoxyglucose positron emission tomography/computed tomography (FDG PET/CT) identified a hypermetabolic lesion limited to the left testis, with no evidence of additional metastases. Choline PET confirmed localized uptake (Figure 2). Prostate‐specific membrane antigen positron emission tomography/computed tomography (PSMA PET/CT) was not performed due to its limited local availability.

**Figure 1:**
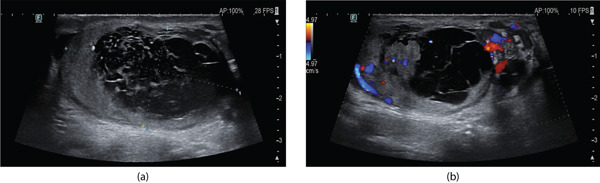
(a,b) Ultrasound image of the left testicle, demonstrating a complete replacement by a solid, hypervascular mass.

**Figure 2:**
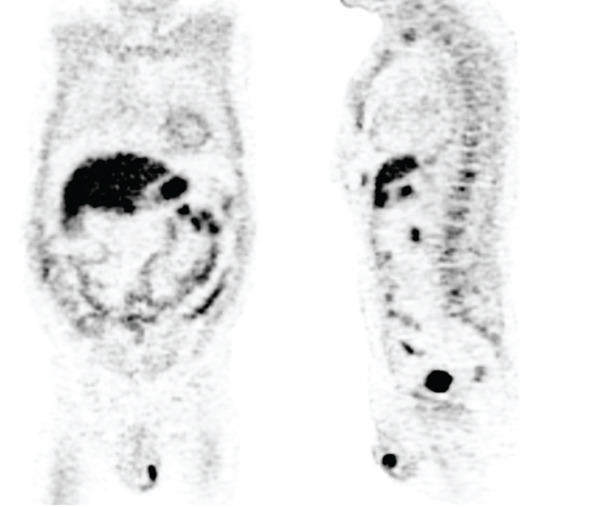
Choline PET/CT demonstrating focal tracer uptake in the left testis, consistent with a metabolically active lesion.

In April 2024, the patient underwent left inguinal orchiectomy. Histological evaluation revealed metastatic carcinoma with papillary architecture consistent with ductal adenocarcinoma of prostatic origin, involving the ipsilateral epididymis. Immunohistochemistry was negative for ERG, PAX8, WT1, and CK7, and positive for PSA, NKX3.1, and CKAE1/AE3 (Figure 3).

**Figure 3:**
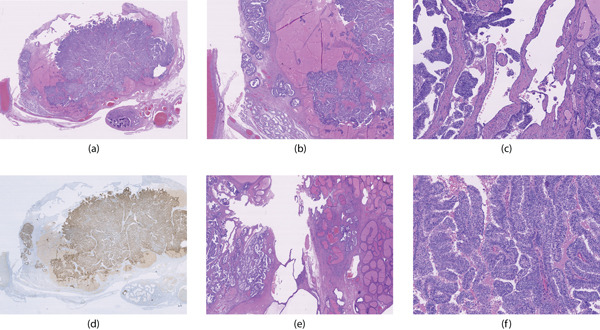
Histopathological images of the left testis. Panels (a, b, c, e, and f) show infiltration of testicular parenchyma and ipsilateral epididymis by ductal prostate carcinoma cells, highlighted with Hematoxylin and Eosin staining. Panel (d) demonstrates NKX3.1‐positive ductal adenocarcinoma cells within the testicular tissue.

Forty days postoperatively, the PSA had decreased to 0.07 ng/mL. After multidisciplinary consultation, a conservative approach without further treatment was adopted. At 12‐month follow‐up, PSA remained undetectable, and the patient was clinically disease‐free. The complete chronological trend of PSA values is summarized in Table 1 and Figure 4.

**Table 1:** Biochemical trajectory from initial treatment to metastatic recurrence.

Date	PSA (ng/mL)	Clinical event
2018 (pre‐op)	6.0	Diagnosis of ductal prostate cancer
2018 (post‐op)	< 0.01	Radical prostatectomy; no adjuvant therapy
2019–2022	< 0.01 (persistently undetectable)	Routine surveillance
March 2023	0.5	CT and pelvic MRI negative (no scrotal imaging)
May 2023	0.6	—
September 2023	2.9	—
November 2023	3.3	—
March 2024	5.6	Left testicular enlargement; imaging confirmed isolated lesion
April 2024	—	Left inguinal orchiectomy
May 2024 (~40 days)	0.07	Postoperative PSA decline
12 months post‐op	< 0.01	Disease‐free

**Figure 4:**
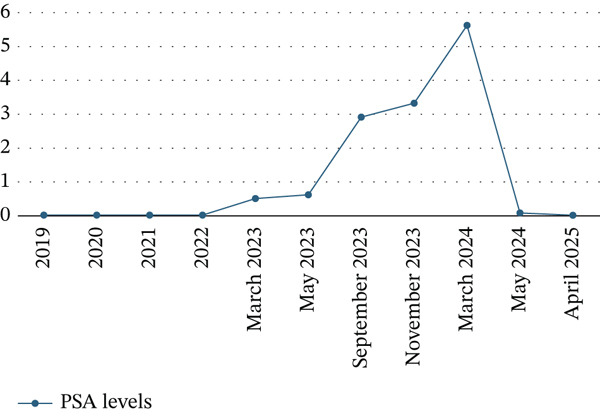
Biochemical trajectory from initial treatment to metastatic recurrence.

## 3. Discussion

Testicular metastases from PCa are exceedingly rare, with an estimated incidence ranging from 0.06% to 0.5% in autopsy series [13, 14] and up to 4% in patients undergoing bilateral orchiectomy for surgical castration [15]. Prostate adenocarcinoma is nonetheless the most frequent solid tumor to metastasize to the testis, excluding hematologic malignancies [14, 16–18].

The pathogenesis of testicular metastases remains speculative. Proposed routes include arterial embolization, retrograde venous or lymphatic dissemination, and endocanalicular spread via the vas deferens [19, 20]. Despite the testis′s rich vascular and lymphatic network, several anatomical and physiological barriers likely contribute to its resistance to metastatic colonization. These include the scrotum′s low‐temperature environment, which may impair tumor cell survival and proliferation, and the blood‐testis barrier, formed by Sertoli cells, peritubular myoid cells, and endothelial tight junctions, which creates an immune‐privileged microenvironment [10–12].

Most published cases of testicular metastasis from PCa involve conventional acinar adenocarcinoma. Cases arising from PDA are far rarer. To date, only six other cases of testicular metastasis from PDA have been documented in the literature up to April 2025 [6–9]. In five of these cases, the primary tumors were classified as high‐risk, with Gleason scores ranging from 8 to 9, whereas Gleason grading was not reported in one case. Additionally, only one other patient presented with a solitary testicular metastasis in the absence of disseminated disease, making the present case the second such report.

PDA is the most common histological variant of PCa, with a reported incidence between 0.15% and 7% [1, 2]. It is considered more aggressive than its acinar counterpart, often presenting at advanced clinical stages and with a higher likelihood of metastasis [3–5].

Although PDA is typically regarded as a high‐grade variant, its clinical behavior may not always parallel conventional Gleason grading. The presence of ductal differentiation itself has been associated with atypical metastatic dissemination patterns independent of initial pathological risk classification [3–5, 21, 22].

Compared with acinar adenocarcinoma, PDA more frequently spreads to atypical sites such as the penis, skin, brain, and testes [6, 21, 22]. Patients with PDA are significantly more likely to present with locally advanced disease (T3–T4) and distant metastases [23, 24]. Testicular involvement, although rare, should thus be considered a possible manifestation of advanced PDA.

The prognosis of isolated testicular metastasis remains unclear due to its rarity and limited clinical data. However, some reports suggest that this rare metastatic pattern may exhibit a limited potential for systemic dissemination and could be associated with more favorable clinical outcomes when compared with other metastatic presentations [25, 26].

The identification of testicular metastasis represents a diagnostic challenge, primarily due to its exceptional rarity and the frequently nonspecific nature of its clinical manifestations. Scrotal ultrasound remains the first‐line imaging modality, whereas functional imaging techniques such as FDG‐PET and choline PET provide further confirmation of testicular involvement and systemic staging.

Although PSA remains the cornerstone biomarker in PCa, its usefulness in PDA is limited due to reduced expression in this variant. PSA levels may not reliably reflect tumor burden in PDA, underscoring the importance of clinical correlation and imaging [27–29].

In our case, however, PSA elevation preceded the clinical manifestation by several months and served as the first indicator of disease recurrence. This observation supports that PSA expression in PDA may be heterogeneous and stage dependent. Although reduced PSA expression has been reported in PDA, serum PSA elevation may still serve as an early indicator of disease recurrence in selected patients.

Prostate‐specific membrane antigen‐positron emission tomography/computed tomography (PSMA‐PET/CT) is superior to conventional imaging techniques (CT, MRI, and bone scans) for detecting metastatic PCa, even at lower PSA levels [30]. However, recent studies have highlighted the limitations of PSMA‐PET/CT in PDA, likely due to the reduced PSMA expression in PDA compared with PAA [27–29]. Additionally, increased GLUT1 expression, particularly in the cell membrane, is characteristic of PDA, suggesting that targeting GLUT1 could offer a novel therapeutic avenue for PDA treatment [31]. The substantial cost of PSMA‐PET/CT limits its routine clinical applicability, drawing attention to the need for more accessible and reliable biomarkers for the diagnosis and monitoring of PDA.

Orchiectomy in cases of testicular metastasis plays both a diagnostic and therapeutic role. In our patient, the procedure led to a significant decrease in PSA levels and sustained remission without the need for additional systemic treatment. Although improved oncological outcomes following radical prostatectomy in PDA have been reported [32, 33], the impact of orchiectomy on survival in cases of isolated testicular metastasis remains undefined and requires further investigation.

Although testicular metastasis from PCa is generally associated with poor prognosis and a median survival of less than 1 year [20, 34, 35], isolated testicular involvement—particularly in the absence of other metastatic sites—may represent a more indolent disease course [25, 26].

Whether adjuvant systemic treatment is warranted postorchiectomy in cases of isolated metastasis remains unclear. Some authors advocate for proactive intervention to prevent further dissemination [36], whereas others favor active surveillance in the absence of other radiologic or biochemical indicators of progression. In our case, a conservative approach was adopted following multidisciplinary evaluation, with excellent short‐term outcomes.

At 13 months, remission is maintained, though the follow‐up duration remains too short to draw meaningful conclusions about long‐term disease control.

## 4. Conclusion

Testicular metastasis from ductal PCa is an exceptionally rare event, with solitary involvement being even more uncommon. This case reinforces the critical role of PSA monitoring as rising levels may precede clinical manifestations of metastasis to atypical sites such as the testis. The absence of scrotal imaging in the initial work‐up delayed the identification of the metastatic site, suggesting that dedicated scrotal evaluation should be considered when PSA rises without a localizable source on standard imaging. Although testicular metastasis often raises concerns about systemic disease, our experience suggests that in the absence of other metastatic lesions, surgical intervention—such as inguinal orchiectomy—can yield a favorable clinical and biochemical response, with sustained remission and no need for additional systemic therapy. This case suggests that isolated testicular metastasis from PDA may follow a less aggressive clinical trajectory than other metastatic patterns and calls attention to the importance of an individualized approach in the management of patients with metastatic PCa.

## Author Contributions

The author takes full responsibility for this article.

## Funding

Open access publishing facilitated by Fondazione Poliambulanza Istituto Ospedaliero, as part of the Wiley—SBBL agreement.

## Disclosure

All authors have read and approved the final version of the manuscript. Martina Romani had full access to all of the data in this study and takes complete responsibility for the integrity of the data and the accuracy of the data analysis.

## Consent

No written consent has been obtained from the patients as there is no patient identifiable data included in this case report.

## Conflicts of Interest

The authors declare no conflicts of interest.

## Data Availability

The data that support the findings of this study are available on request from the corresponding author.
